# Exertional heat stroke-induced changes in gut microbiota cause cognitive impairment in mice

**DOI:** 10.1186/s12866-024-03276-7

**Published:** 2024-04-23

**Authors:** Jiangang Xie, Linxiao Wang, Yunyun Xu, Yuexiang Ma, Lingqin Zhang, Wen Yin, Yang Huang

**Affiliations:** 1 Department of Interventional Vascular, Xi’an No.3 Hospital, the Affiliated Hospital of Northwest University, Xi’an, Shaanxi Province 710018 China; 2 College of Life Sciences, Northwest University, Xi’an, 710127 China; 3 Department of Emergency, Xijing Hospital, Air Force Medical University, Xi’an, Shaanxi Province 710000 China

**Keywords:** Exertional heat stroke, Gut microbiota, Gut-brain axis, Probiotic, Cognitive impairment

## Abstract

**Background:**

The incidence of exertional heat stroke (EHS) escalates during periods of elevated temperatures, potentially leading to persistent cognitive impairment postrecovery. Currently, effective prophylactic or therapeutic measures against EHS are nonexistent.

**Methods:**

The selection of days 14 and 23 postinduction for detailed examination was guided by TEM of neuronal cells and HE staining of intestinal villi and the hippocampal regions. Fecal specimens from the ileum and cecum at these designated times were analyzed for changes in gut microbiota and metabolic products. Bioinformatic analyses facilitated the identification of pivotal microbial species and metabolites. The influence of supplementing these identified microorganisms on behavioral outcomes and the expression of functional proteins within the hippocampus was subsequently assessed.

**Results:**

TEM analyses of neurons, coupled with HE staining of intestinal villi and the hippocampal region, indicated substantial recovery in intestinal morphology and neuronal injury on Day 14, indicating this time point for subsequent microbial and metabolomic analyses. Notably, a reduction in the *Lactobacillaceae* family, particularly *Lactobacillus murinus*, was observed. Functional annotation of 16S rDNA sequences suggested diminished lipid metabolism and glycan biosynthesis and metabolism in EHS models. Mice receiving this intervention (EHS + probiotics group) exhibited markedly reduced cognitive impairment and increased expression of BDNF/TrKB pathway molecules in the hippocampus during behavioral assessment on Day 28.

**Conclusion:**

Probiotic supplementation, specifically with *Lactobacillus spp.*, appears to mitigate EHS-induced cognitive impairment, potentially through the modulation of the BDNF/TrKB signaling pathway within the hippocampus, illustrating the therapeutic potential of targeting the gut-brain axis.

**Supplementary Information:**

The online version contains supplementary material available at 10.1186/s12866-024-03276-7.

## Information

Heat stroke constitutes a critical medical emergency characterized by a core body temperature exceeding 105 °F (40.5 °C) and central nervous system impairment [1]. It is categorized into exertional heat stroke (EHS) and classic (non-exertional) heat stroke [2]. The pathophysiological alterations in EHS encompass protein denaturation, endotoxemia, and failure of thermoregulation, leading to a systemic inflammatory response syndrome akin to septic shock, which can precipitate multiorgan impairment and mortality [3]. Acute hyperthermia from EHS typically presents with neuropsychiatric symptoms, ranging from cognitive impairment to seizures and consciousness disturbances, potentially progressing to lethargy or coma [4]. Hyperthermia can inflict significant neuronal damage, affecting electrochemical depolarization, ion transport, signaling pathways, and mitochondrial function [5]. Neurological sequelae from EHS, a primary contributor to heatstroke-induced fatality, can lead to persistent neurological and cardiovascular disorders, elevating long-term mortality risk. Currently, the mainstay of EHS management is prompt cooling [6]. While the immediate effects of EHS are well recognized, the long-term health consequences warrant further investigation. Studies indicate that all individuals with heat stroke present acute neurological symptoms, and approximately 4.4% of EHS survivors sustain permanent neurological damage, predominantly manifesting as motor or cognitive impairments [7]. Despite the profound impact of EHS-induced cognitive impairment, research in this domain remains sparse.

The gastrointestinal system is highly susceptible to heat-induced injury during EHS [8]. Heat stress from EHS provokes hyperthermia and oxidative stress within the gut epithelium, compromising barrier integrity, enhancing infection risk, and causing inflammation, all of which alter the gut microbiome's composition and abundance [9]. The gut, a primary target organ under heat stress, experiences alterations in mucosal architecture, decreased immunity and heightened permeability to endotoxins and pathogens [10]. The gut microbiota modulates brain function and cognition through the microbiota-gut-brain axis, involving metabolic, peripheral immune, and central nervous systems [11–13]. This communication is essential for overall health, and disruptions in this interplay are believed to be significant in the pathogenesis of numerous diseases [13]. Probiotics, or probiotic-driven symbiotic formulations, are posited to offer preventive and therapeutic benefits [14].

The cognitive impairment associated with EHS can be attributed not only to thermal injury to cerebral structures but also to potential disturbances in the gut-brain axis, a topic yet to be extensively documented. The gut microbiota may influence cerebral homeostasis directly or indirectly through the secretion of metabolites that affect mood and cognitive abilities. It has been established that probiotic treatments can adjust gut microbial populations and reinforce gut barrier integrity during EHS [14]. This study was designed to investigate the role of the gut microbiota in emotional and cognitive outcomes post-EHS. Utilizing a murine EHS model, we examined alterations in behavior, microbiota composition, and metabolic profiles by applying bioinformatic and metabolomic approaches. Furthermore, the investigation assessed the involvement of the gut microbiota in managing negative emotions and cognitive functions following EHS and determined the impact of probiotic supplementation on these interactions.

## Methods

### Establishment of animal model

Male C57BL/6 mice, aged 8-10 weeks, were procured from the Animal Center at the Fourth Military Medical University. Prior to experimentation, the animals underwent a week-long acclimatization period within a thermally controlled simulation chamber (Mouse rotary fatigue apparatus, Shanghai, China) at a constant rotational speed of 10 revolutions per minute (*rpm*) under standard ambient temperature and relative humidity (65 ± 5%). Any mice exhibiting abnormal rectal temperatures were excluded from the study. The experimental conditions were set to a stable temperature of 39.5 ± 0.5 ℃ and relative humidity of 65 ± 5%, with the treadmill speed initially set at 10 *rpm* and incrementally increased to a maximum of 17 *rpm*. The mice were diligently monitored for any signs of distress. The induction of EHS was confirmed by assessing the loss of the righting reflex, which is indicative of severe neurological impairment. Following the induction protocol, mice were returned to their standard housing conditions with ad libitum access to food and water [15]. All procedures involving animals were conducted in compliance with the Guide for the Care and Use of Laboratory Animals and received approval from the Institutional Animal Care and Use Committee of the Fourth Military Medical University (IACUC-20230039).

### Behavioral testing

#### Open field test (OFT)

The OFT was administered after allowing the mice a 1-hour habituation period to the testing environment. The apparatus consisted of an open arena measuring 50 cm × 50 cm × 40 cm, with even illumination across the four corners. The arena was segmented into 16 equal squares, with the central four squares delineating the middle section. Each mouse was gently placed at the center of the arena facing away from the investigator and was permitted to explore for a duration of 10 minutes. Movement patterns were videotaped and subsequently analyzed for total distance traveled and time spent in the central zone utilizing Noldus EthoVision tracking software [16].

#### Elevated plus-maze test (EPM)

In preparation for the EPMT, mice underwent a one-week acclimation process involving gentle handling by the investigators for periods ranging from 1 to 5 minutes daily to mitigate anxiety related to human contact. During the test, mice were positioned at the central junction of the maze facing an open arm. Sessions were videotaped, capturing both the frequency and duration of entries into open and closed arms over a 5-minute period. The observer maintained a distance of 1 meter from the maze to avoid influencing the animals' behavior [17].

#### Tail suspension test (TST)

For the TST, mice were suspended by the final third of their tail from a support apparatus, ensuring that the head was elevated 15 centimeters from the surface. The experimental setup incorporated a background contrastive to the coloration of the mice to facilitate clear video recording. After a period of 6 minutes, the duration of immobility was quantified using Smart V3.0 animal behavior analysis software [17].

#### Forced swimming test (FST)

The FST involved a cylindrical container filled with water maintained at 25 ± 1 °C. Water volume was adjusted in accordance with the size of the test subjects. Premarkings on the container aided in postexperimental analysis. Mice were placed into the water promptly to minimize stress, and their behavior was recorded over a 6-minute interval using Smart V3.0 software for automated activity tracking [17].

#### Novel object recognition test (NORT)

The NORT consisted of three stages: habituation, training, and testing. During habituation, mice explored an open arena for 10 minutes on the initial day. On a subsequent day, training involved 10 minutes of exposure to two identical objects centrally located within the arena. For the test phase, each mouse was presented with one familiar object (A1) and one novel object (A2) and monitored for 10 minutes. Exploration was defined as touching the object or directing the head toward it within a 2 cm proximity. All objects and surfaces were sanitized with 75% ethanol before each session. The preference index was calculated as the ratio of the duration of exploration of the novel object (A2) to the combined exploration time for both objects (A1+A2) [18].

### Gut microbiota 16S sequencing of EHS mice

The 16S rRNA gene sequencing of the gut microbiota from EHS mice was conducted using the Illumina NovaSeq 6000 system (Gene Denovo Biotechnology Co., Ltd, Guangzhou, China). DNA was extracted from fecal samples utilizing the HiPure Stool DNA Kit (Magen, Guangzhou, China). Amplification of the 16S rDNA V4-V5 regions was performed via PCR, followed by purification from a 2% agarose gel and subsequent quantification. The amplicons were then sequenced on the Illumina platform, adhering to the manufacturer's protocol for paired-end reads (PE250). Raw sequences were processed to filter out low-quality reads, and the remaining high-quality sequences were merged into tags. These tags were clustered and chimera-filtered to yield accurate taxonomic abundance data [19]. Representative sequences for each operational taxonomic unit (OTU) were classified using the RDP Classifier with a Bayesian algorithm at a confidence level of 0.8. Bioinformatics analysis was facilitated by the Omicsmart platform.

### Sequencing of gut microbiota in EHS mice

Metabolomic profiling was conducted using an Agilent 1290 Infinity LC system coupled with Q Exactive Orbitrap mass spectrometry (Thermo Fisher Scientific). Separation was achieved on an HSS T3 column. The mobile phase for positive electrospray ionization (ESI) mode consisted of 0.1% formic acid in water (A) and acetonitrile (B), whereas for negative ESI mode, 0.5 mM ammonium fluoride in water (A) and acetonitrile (B) were used. Source conditions for the ESI were set with a sheath gas flow rate of 45 arbitrary units, auxiliary gas flow rate of 15 arbitrary units, capillary temperature of 320 ℃, full MS resolution of 70,000, and MS/MS resolution of 17,500. The normalized collision energy was set at 20, 40, and 60 eV. The spray voltage was adjusted to 3.8 kV for positive and -3.1 kV for negative modes. The gradient profile began with 1% B, increased linearly to 99% B over 11.5 minutes, held for 3.5 minutes, and then returned to 1% B over 0.1 minutes, with a re-equilibration period of 3.4 minutes. The column temperature was maintained at 25 ℃ with a flow rate of 0.3 mL/min. The injection volume for the samples was 2 μL. Bioinformatic analyses were employed to identify potential metabolites, with selection criteria including variable importance in projection (VIP) score exceeding 1.

### Multivariate analysis

Orthogonal partial least squares (O2PLS) multivariate analysis was conducted to delineate the associations between the 16S rDNA regions and the metabolomic profiles. Data preprocessing, including normalization via ultraviolet (UV) or Pareto scaling (Par), facilitated the distinction of differences between groups. O2PLS modeling and Spearman's rank correlation analysis were carried out using SIMCA software version 14.1 (Sartorius Stedim Biotech, Göttingen, Germany). This bidirectional modeling approach, capable of handling multiple dependent variables, yielded a model with robust predictive accuracy and reliability, as evidenced by R2Y values equal to or exceeding 0.65 and Q2 values of 0.5 or higher.

### Behavioral effects of probiotic supplementation in EHS mice

Female C57BL/6 mice, aged 6-8 weeks and weighing 20-25 grams, were assigned to one of three groups: control (*n* = 6), EHS (n = 12), or EHS with daily probiotic supplementation postinduction (EHS + probiotics, *n* = 12). The probiotic blend administered to the EHS + probiotics group comprised strains including *Bifidobacterium bifidum* F-35, *Bifidobacterium longum* CCFM729, and several *Lactobacillus* species at a dose of 0.02 mg/kg. Control and EHS mice received galacto-oligosaccharide at a concentration of 20.2% [15–18]. Behavioral assessments, specifically the open field test (OFT) and novel object recognition test (NORT), were conducted one day prior to and 28 days following EHS model establishment.

### Western blot

Hippocampal proteins were extracted from murine tissue, quantified using the bicinchoninic acid (BCA) assay post ultracentrifugation, denatured, and subjected to SDS‒PAGE. Following electrophoresis, proteins were transferred to polyvinylidene difluoride (PVDF) membranes. The membranes were blocked briefly and incubated overnight at 4 °C with primary antibodies against BDNF, TrkB (Cell Signaling Technology #ab108319), phosphorylated TrkB (Bioss Antibodies #bsm-52213R), and β-Actin (Cell Signaling Technology #4967) [20]. Subsequent washes with Tris-buffered saline containing 0.1% Tween 20 (TBST) preceded incubation with secondary antibodies for 1 hour. Chemiluminescent detection was conducted using the Western Lumax Light system (Zeta Life), with imaging and densitometric analysis performed on the Chemi Doc MP system (Bio-Rad, Hercules, CA, USA).

### Statistical analysis

Research execution adhered to randomization and blinding principles to ensure objectivity. The results are presented as the mean ± standard error of the mean (SEM). Statistical evaluations were carried out using both one-way and two-way analysis of variance (ANOVA) via GraphPad Prism version 8. A *p* value less than 0.05 was considered indicative of statistical significance.

## Results

### Behavioral tests in EHS mice suggested cognitive impairment

**Fig. 1:**
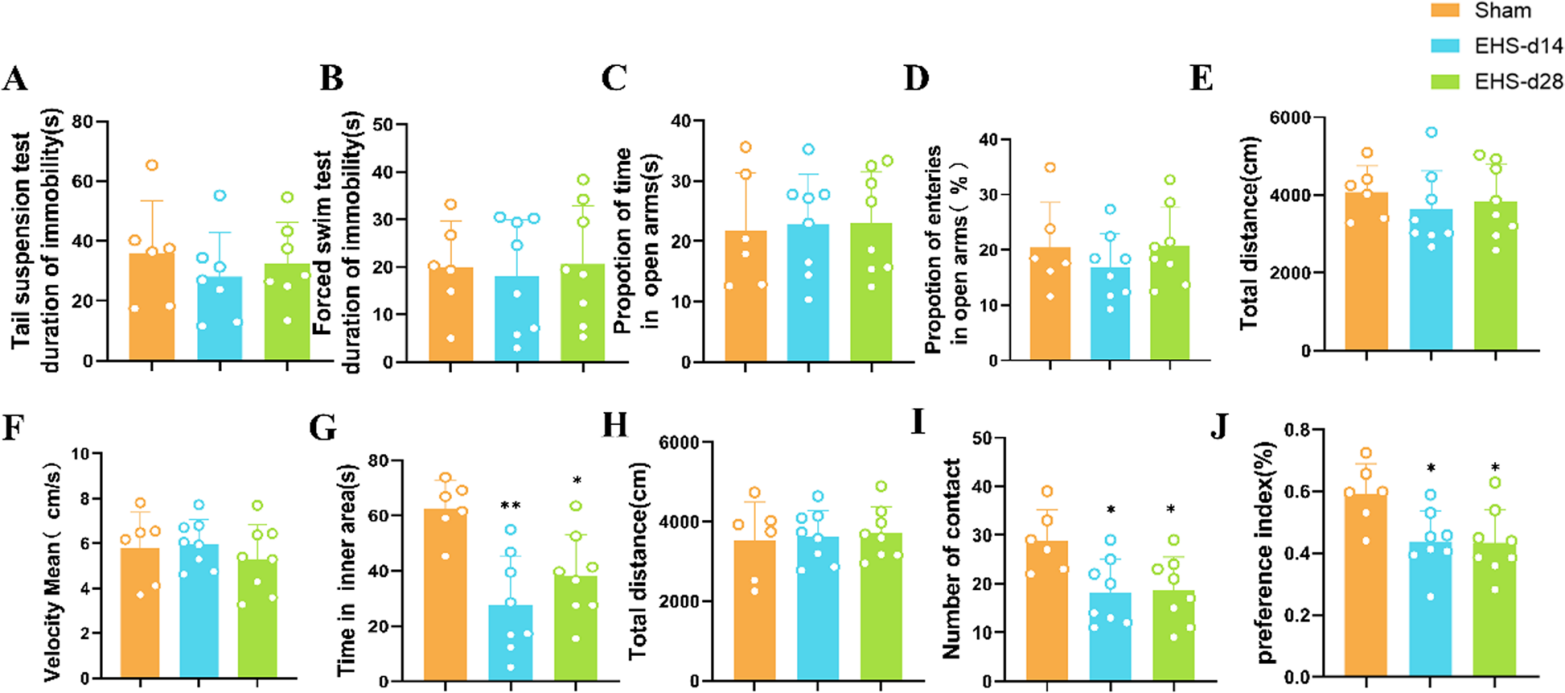
Behavioral testing of EHS mice models. **A** TST and **B** FST reveal markedly depressive-like behavior in mice. **C** and **D** show no significant change in EPMT; **E** and **F** show no difference in EPMT; **G** show the time the mice were in the inner region. **H**, **I** and **J** are NORT experiments. Data are expressed as the mean ± SEM (sham, *n* = 6; EHS 14 d, *n* = 12; EHS 28 d, *n* = 12). ^*^p < 0.05, ^**^p < 0.01, ns = no statistical significance. *vs*. sham. An unpaired T test was used for data analysis

In the TST and FST, the EHS cohort did not exhibit significant signs of depressive-like behavior compared with the control group at various time points (Fig. 1A and B). Similarly, evaluations using the elevated plus-maze test (EPMT) revealed no substantial alterations in the number of entries or the time spent in the open arms by EHS mice on Days 14 and 28 compared to controls (Fig. 1C and D). The OFT, a measure of autonomous and exploratory behaviors as well as anxiety levels in novel environments, revealed no appreciable differences in locomotor activity between groups, as indicated by travel distance and velocity on Days 14 and 28 (Fig. 1E and F). However, a reduced duration spent in the central area was noted on Day 14 (Fig. 1G). The NORT highlighted a discernible reduction in the interaction with novel objects, time engaged with these objects, and cognitive indices (e.g., preference indices) following a 14-day interval (Fig. 1I and J), with no variance in total movement (Fig. 1H). Collectively, these findings imply that EHS mice do not present with affective disturbances such as anxiety or depression, yet they demonstrate notable cognitive impairment.

### Normalization of intestinal and nerve injury in the hippocampal region of the brain in EHS mice

**Fig. 2:**
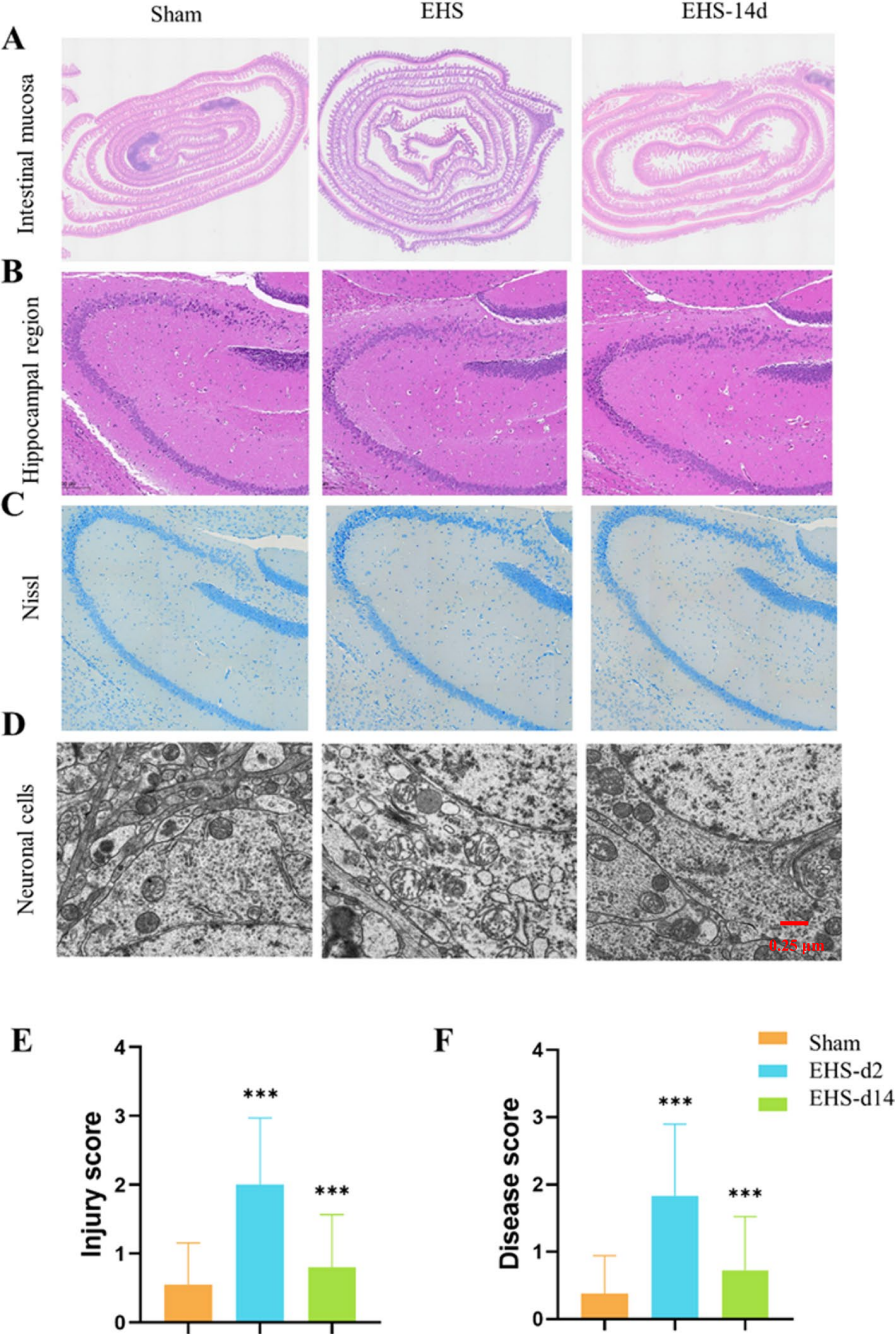
Intestinal villi and hippocampal region injury in EHS mice. **A** HE staining of intestinal villi (×100); **B** HE staining of hippocampal region (×100); **C** Nissl staining of hippocampal region (×100); **D** TEM of neurons in the hippocampal region; **E** Chiu’s scale for evaluating the degree of intestinal injury; **F** The Flameng scale was used to evaluate the degree of neuronal mitochondrial injury. Data are expressed as the mean ± SEM (*n* = 12/group). ^***^p < 0.001, ns = no statistical significance. *vs*. sham. Unpaired. A *t* test was used for data analysis

In the EHS mice, cognitive impairment became apparent by Day 14, yet no prior research has elucidated the association with potential intestinal and hippocampal injury. To investigate this, hematoxylin and eosin (HE) staining was conducted on the intestinal villi and hippocampal neurons. Moreover, neuronal structure was examined via transmission electron microscopy (TEM). HE staining of the EHS mice revealed pronounced intestinal damage, characterized by enlargement and distortion of villi, separation of the lamina propria, and capillary dilation with increased cellular infiltration within the lamina propria. Notably, minimal digestion and disintegration of the lamina propria were observed. By Day 14, these alterations had resolved, and intestinal villi injury scores were comparable to those of the control group (Fig. 2A and E). Pathological assessment of the hippocampus through HE and Nissl staining indicated that control animals exhibited neurons with distinct nuclei and orderly cell arrangement. In contrast, EHS mice demonstrated pronounced nuclear pyknosis and indistinct nuclei in dentate gyrus (DG) granule cells, which were notably diminished by Day 14 (Fig. 2B). Nissl staining revealed a decrease in Nissl bodies, disorganized cellular architecture, and neural damage in the EHS group, which also returned to baseline by Day 14 (Fig. 2C). TEM analysis of neuronal mitochondria within the EHS cohort showed swelling, a clear matrix, and some disrupted cristae. These structural changes were significantly ameliorated by Day 14 (Fig. 2D and F).

### Variations in *Lactobacillus* murinus of the genus in EHS mice

**Fig. 3:**
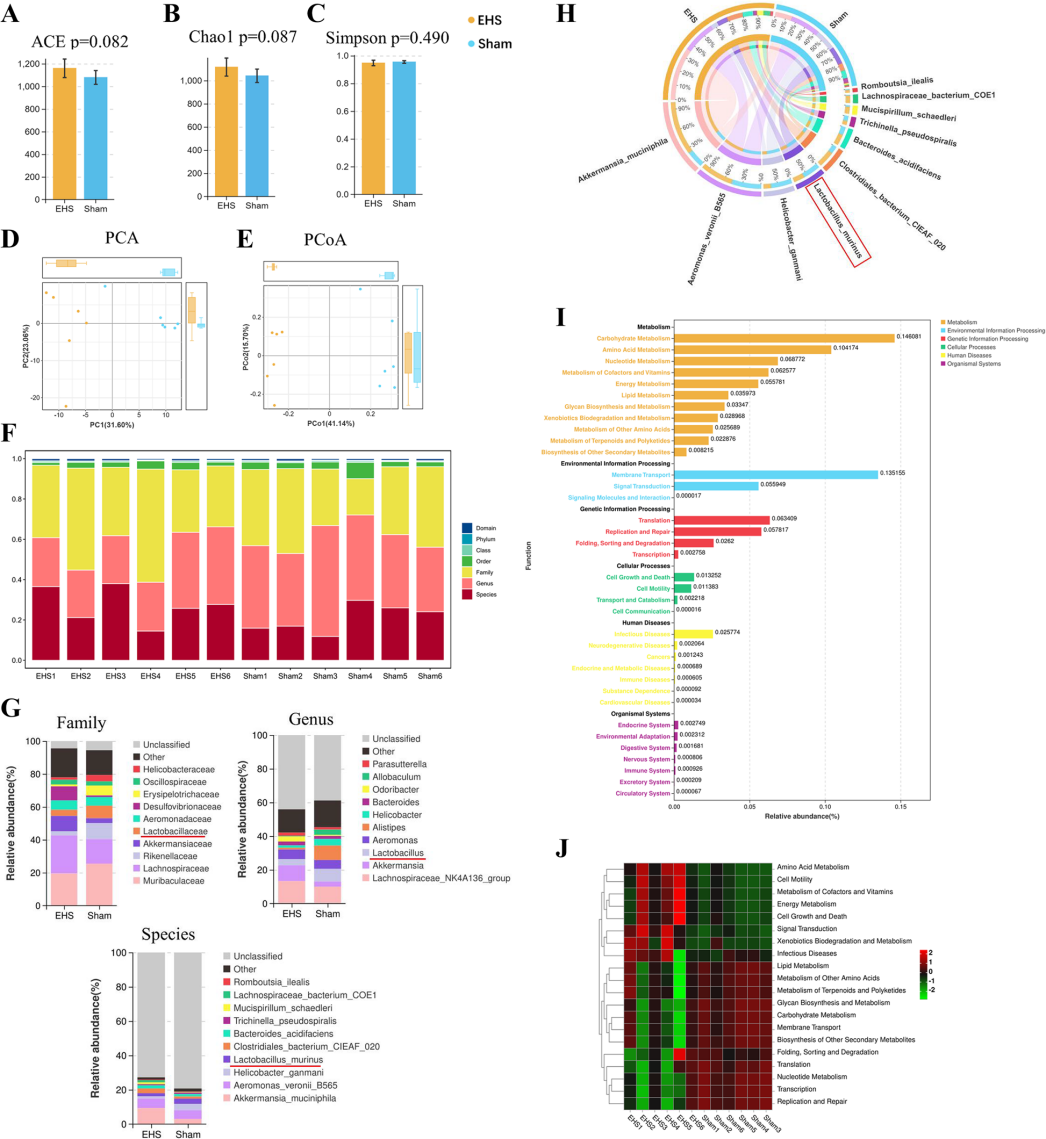
Gut microbiota 16S sequencing of EHS mice. **A-C** α diversity of ACE, Chao1, and Simpson indices of the gut microbiota in the two groups. **D-E** β diversity analysis of the samples in the two groups at the PCA and PCoA level. **F** Waterfall plot of EHS mice. **G** Distribution stacked map of family, genus, species. **H** Distribution circle map of 16S species composition. **I** PICRUSt2 Functional Distribution General Map. **J** Heatmap for metabolite sequencing of the gut microbiota of sham group and EHS group mice

Subsequent to the observation of HE staining and the evaluation of neuronal integrity via TEM, recovery of intestinal and hippocampal damage was noted in EHS mice on Day 14 post-event. To elucidate the microbiota changes, fecal samples from the sham (*n* = 6) and 14-day post-EHS (*n* = 12) mice were subjected to high-throughput sequencing to analyze the intestinal microbiome composition. The sequencing data indicated that the microbial diversity (α-diversity) remained constant, whereas the microbial community structure (β-diversity) exhibited alterations post-EHS (Fig. 3A, C, D, and E). Distinct clustering of microbial taxa at the family, genus, and species levels was discerned in the EHS mice, as shown in the microbiota waterfall plot (Fig. 3F). A notable decrease in the abundance of the family *Lactobacillaceae*, specifically in the genus and species *Lactobacillus murinus*, was observed (Fig. 3G and H). Further analysis using PICRUSt2 for functional predictions demonstrated that EHS influenced various biological processes in the gut microbiota, including amino acid and lipid metabolism, replication and repair mechanisms, cellular growth and death, membrane transport, and signal transduction (Fig. 3I). The heatmap of functional abundance highlighted a significant reduction in pathways associated with amino acid metabolism, cell motility, cellular growth and death, signal transduction, xenobiotic biodegradation and metabolism, metabolism of cofactors and vitamins, and energy metabolism following EHS (Fig. 3J).

### EHS significantly altered the metabolites of the gut microbiota

**Fig. 4:**
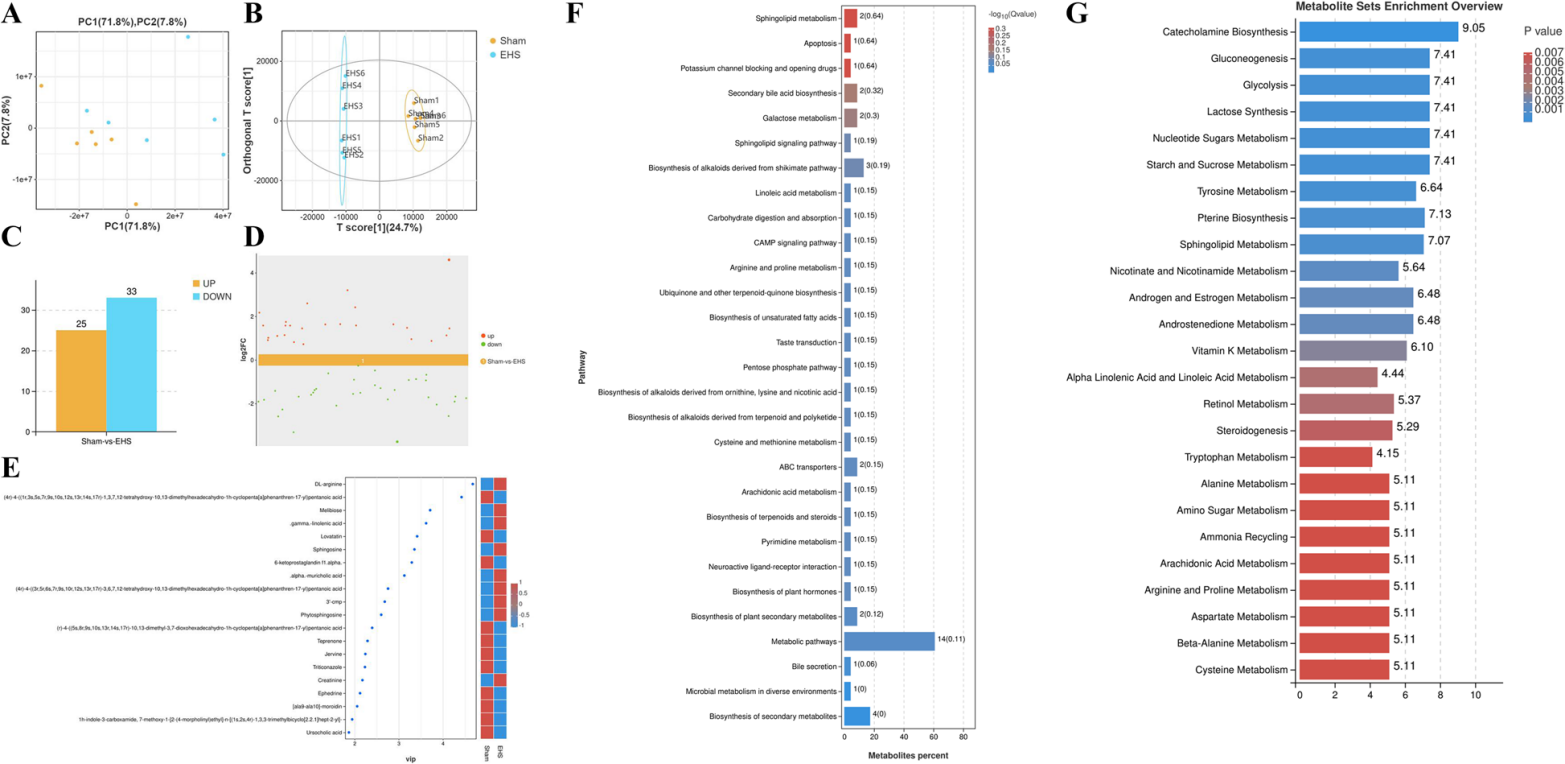
Gut microbiota metabolite sequencing analysis of sham and EHS mice. **A** PCA score plot of the sham and EHS groups; **B** orthogonal T scores of the sham and EHS groups; **C** and **D** differential expression of metabolites in the EHS and sham groups; **E** cluster heatmap of the differentially expressed metabolites between the two groups; **F** KEGG functional enrichment analysis of the differentially expressed metabolites; **G** MSEA of the differentially expressed metabolites

An investigation was conducted to ascertain the influence of EHS on the metabolomic profile of the intestinal microbiota. Fecal samples from the control (sham, *n* = 6) and EHS-affected mice on Day 14 (*n* = 12) were subjected to metabolomic sequencing. Principal component analysis (PCA) and orthogonal projections to latent structures (O2PLS) demonstrated distinct, statistically significant differences between the sham and EHS groups, confirming the robustness of the analytical methods employed (Fig. 4A and B). A total of 58 differentially expressed metabolites were identified in the EHS group, comprising 25 that were upregulated and 33 that were downregulated relative to the sham group (Fig. 4C and D). Heatmaps clustering these metabolites highlighted disruptions in the metabolism of short-chain fatty acids (SCFAs) and sphingosine, with pentanoic acid being particularly elevated (Fig. 4E). Enrichment analysis using the Kyoto Encyclopedia of Genes and Genomes (KEGG) and metabolite set enrichment analysis (MSEA) showed that the altered metabolites were predominantly involved in pathways such as sphingolipid metabolism, linoleic acid metabolism, biosynthesis of unsaturated fatty acids, arachidonic acid metabolism, catecholamine biosynthesis, and the metabolism of tyrosine, arginine, proline, alanine, and aspartate (Fig. 4F and G).

### Correlation between *Lactobacillus* murinus and metabolite levels in the gut microbiota of mice

**Fig. 5:**
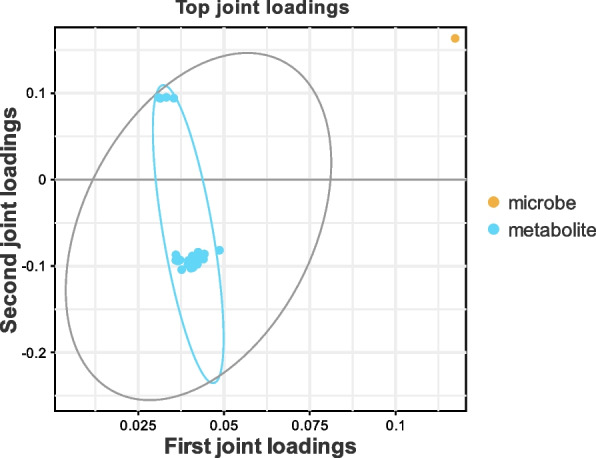
Metabolites associated with the O2PLS model *Lactobacillus*

To delve deeper into the metabolic alterations associated with dysbiosis in the gut microbiota of EHS mice, a Spearman correlation analysis was conducted. This analysis revealed linkages between 13 differentially abundant microbial species and the levels of 80 distinct metabolites in serum. Notably, two species within the *Lactobacillus* genus demonstrated significant correlations with altered metabolite concentrations, as determined through O2PLS association analysis (Fig. 5).

### Probiotics improve cognitive impairment by promoting the BDNF/Trkb pathway

**Fig. 6:**
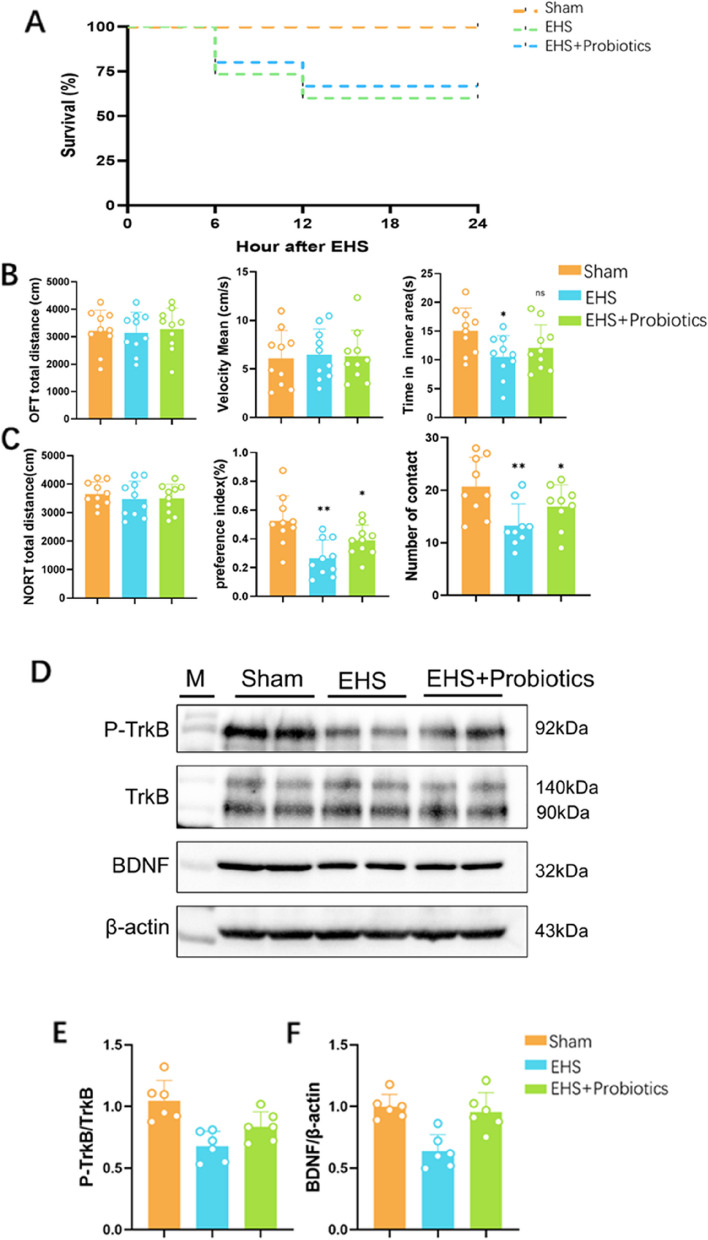
Influence of probiotic supplementation on behavioral and mechanistic effects in mice. **A** Survival curves of different groups of mice; **B** Distance and time to center area of OFT; **C** Detect the exploration time and preference index of NORT; **D** Western blot for protein expression of BDNF, TrkB and p-TrkB; **E** Quantification of BDNF protein expression; **F** Quantification of the p-TrkB/TrkB protein ratio. Data are expressed as the mean ± SEM (*n* = 12 group). ^*^p < 0.05, ^**^p < 0.01, ^***^p < 0.001, ns = no statistical significance. Data are expressed as the mean ± SEM (*n* = 6 group)

Investigations into the gut-brain axis revealed *Lactobacillus* and associated metabolites as potential modifiers in cognitive impairment [21]. Probiotic formulations, pioneered at Jiangnan University, were administered to the EHS mice, resulting in no observed increase in mortality within the initial 12 hours posttreatment (Fig. 6A). Subsequent behavioral assessments, including the OFT and NORT, indicated a probiotic-mediated enhancement in cognitive performance (Fig. 6B and C). Protein assays in hippocampal tissues corroborated these findings, showing that probiotic treatment upregulated the expression of BDNF and its receptor, TrkB, along the gut-brain axis (Fig. 6D and F). The details of Fig. D show in WB Raw date.

### Mechanism

**Fig. 7:**
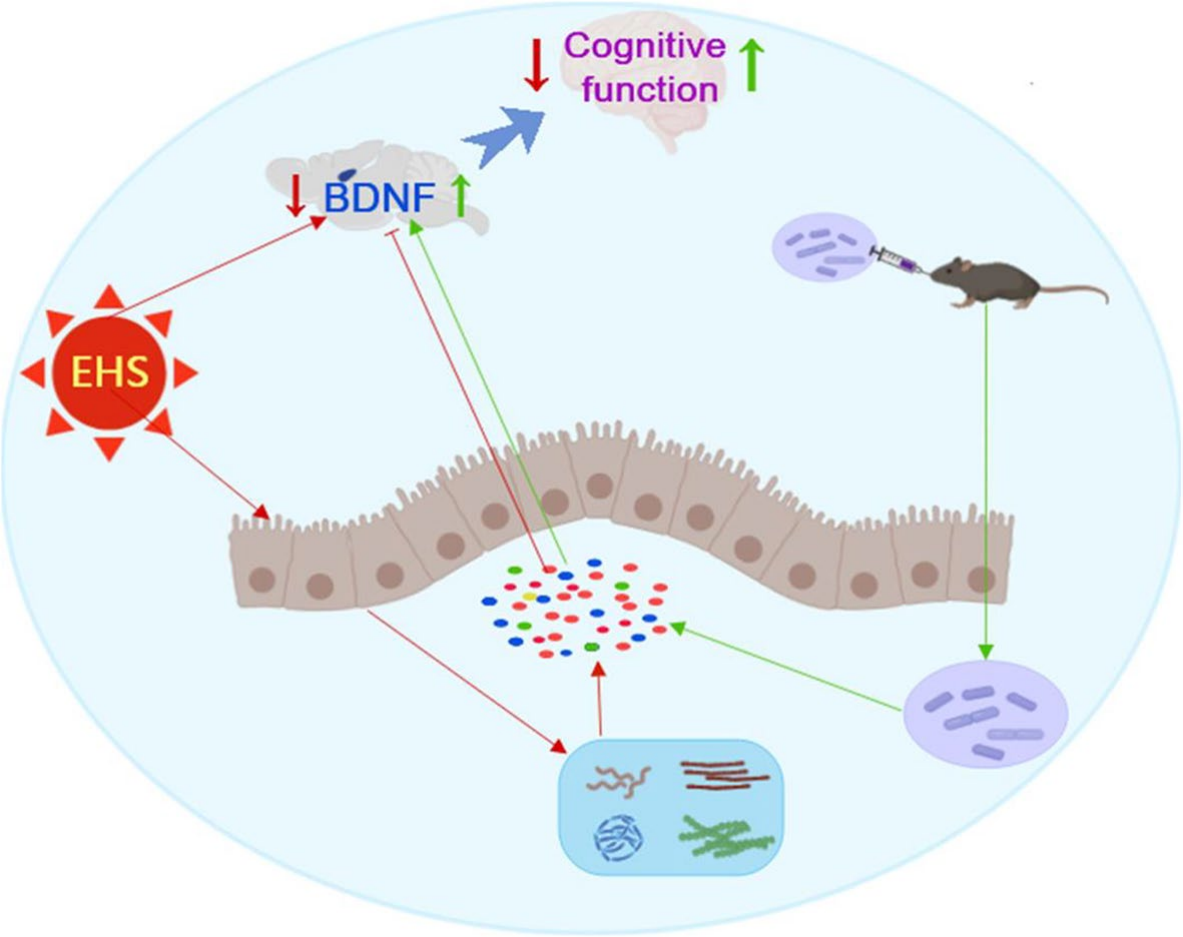
Hypothesis mechanism diagram of EHS mice. Reduced *Lactobacillus* and abnormal short-chain fatty acid metabolism promote cognitive impairment due to heat injury in EHS. Supplementation with probiotics improved dysbiosis and promoted the expression of the BDNF/TrKB pathway in the cortex and hippocampus via the gut-brain axis, alleviating cognitive impairment

Heat injury from heat stroke disrupts gut microbiota equilibrium, precipitating a decline in *Lactobacillus* populations and the subsequent dysregulation of short-chain fatty acid (SCFA) metabolism. These events are implicated in the emergence and maintenance of cognitive impairment. The administration of *Lactobacillus* restores microbial homeostasis and facilitates recovery from cognitive impairments by enhancing the BDNF/TrkB signaling pathway within cortical and hippocampal regions through gut-brain axis interactions (Fig. 7).

## Discussion

Prior research has demonstrated that EHS leads to direct thermal injury of cerebral tissues, resulting in sustained damage characterized by motor impairment, particularly cerebellar impairment, alongside affective and cognitive impairment [22]. Subsequent investigations have thus been oriented toward identifying cerebral protective mechanisms post-pathogenesis, with strategies proposed to attenuate such sequelae through the modulation of complex genetic signaling pathways [23, 24]. However, the translation of these methods into clinical practice remains a challenge. The gastrointestinal system, which is notably vulnerable, undergoes mucosal injury, increased permeability, and microbiota disturbances in response to EHS. This condition is implicated in altering the gut microbiota's composition, in turn predisposing the gut to heightened stress-related damage [25]. The dysbiosis associated with EHS is linked to neurobehavioral changes, suggesting that therapeutic modulation of the gut microbiota may offer a practical strategy for clinical translation. Our research concentrates on the post-EHS period, particularly examining orthopsychiatric behaviors and their correlation with the gut-brain axis, with the goal of improving EHS patient outcomes through interventions targeting the gut microbiota. The recovery timeline post-heat stroke is approximately 14 days from the start of coma [26]. Observations indicate an absence of anxiety and depression in mice after 28 days of recovery; however, cognitive impairments were noted. Consequently, we selected the 14- and 28-day marks post-EHS to evaluate behavioral changes. The study confirms that EHS results in cognitive impairments and motor impairment due to lasting neurological damage [7]. Mice displaying exercise capacity anomalies were not observed, potentially due to the premature demise of those with severe brain injuries within the initial 12 hours. Nonintervention in EHS-affected mice yielded a concentrated mortality rate of 40% within this time frame, which is consistent with mortality rates reported in other EHS studies [6]. The persistence of cognitive impairment from Day 14 to Day 28 suggests the possibility of earlier cognitive changes post-EHS onset.

In response to heat stress, gut microbiota stimulates gut immune cells to generate and secrete cytokines that induce the generation of an immune response to modulate the brain. In turn, the brain utilizes the same pathways to alter the gut microbiota, especially their composition and abundance [9, 27]. The 14 days after EHS onset, marked alterations were observed in the intestinal microbiota at multiple taxonomic levels, including a pronounced decrease in *Lactobacillus* across family, genus, and species categories. Although *Lactobacillus* represents a minor constituent of the human microbiota, its relative abundance frequently exhibits a positive or negative correlation with human diseases and chronic health conditions. Advances in the study of *Lactobacillus* within human and animal microbiomes, as well as expanding knowledge of probiotics and dietary *Lactobacillus*, have yielded new perspectives on its role in human health regulation [28]. KEGG pathway analysis identified that EHS induced differential enrichment in various biological processes, notably in amino acid metabolism, lipid metabolism, replication and repair, cellular growth and death, membrane transport, and signal transduction. Subsequent examination through functional abundance heatmaps elucidated significant reductions in these biological functions post-EHS, encompassing amino acid metabolism, cell motility, cellular growth and death, signal transduction, xenobiotic biodegradation, metabolism of cofactors and vitamins, and energy metabolism. These findings suggest that *Lactobacillus* and other probiotics may offer therapeutic benefits in addressing microbial-gut-brain axis perturbations, with implications for cognitive impairments associated with aging [29]. Nonetheless, the observed changes in KEGG pathways may not solely result from a decline in *Lactobacillus* levels but could also be a consequence of thermal stress-induced injury to the gut microbiota and subsequent functional modifications.

The gut-brain axis is defined by significant bidirectional communication between the intestinal microbiota and brain tissue, mediated by metabolic byproducts. Our investigation into the metabolic irregularities of short-chain fatty acids (SCFAs) and sphingosine, particularly the predominance of pentanoic acid, involved analysis of Day-14 post-EHS microbiota. Correlations have been observed between fecal SCFA levels and inflammatory markers, as well as compromised cognitive functioning in schizophrenia patients, suggesting a possible role for valeric acid metabolism in memory modulation [30]. Metabolomic analysis via KEGG and MSEA indicated that EHS induces metabolic enrichment in pathways including sphingolipid, linoleic acid, unsaturated fatty acid biosynthesis, arachidonic acid metabolism, and various amino acid metabolism pathways. These metabolites differ significantly from neurotransmitters yet are integral to metabolic pathways. The influence of SCFAs on neuronal health has been substantiated, with evidence indicating that fecal microbiota transplantation (FMT) and SCFA supplementation can mitigate hippocampal damage from stroke by supporting mitochondrial functionality and prompting metabolic adaptation to counteract oxidative phosphorylation impairment [31]. Such findings affirm the pivotal role of SCFAs in the adaptive response of neuronal cells. Investigations into *Lactobacillus rhamnosus* strains revealed that *L. rhamnosus* SD11 exhibits superior butyrate production [32]. Probiotic administration has been shown to enhance SCFA levels, particularly acetate, propionate, and butyrate [33]. Hence, *Lactobacillus* appears to augment SCFAs and associated metabolite production. This study examined the potential benefits of probiotic supplementation in EHS management, considering the interrelationship between intestinal microbiota and metabolism. The literature suggests that probiotics maintain intestinal barrier integrity and mitigate inflammatory responses [14]. In models where *Lactobacillus* was administered post-EHS, outcomes included improved heat resilience, reduced gastrointestinal damage, diminished inflammatory mediator release, and elevated tight junction protein expression [34].

Recent investigations have demonstrated a notable enhancement in cognitive function in mice post-EHS upon administration of *Lactobacillus*, with behavioral assessments conducted on Day 14 indicating improved outcomes. Subsequent research indicated that probiotics supplementation fosters an increase in BDNF protein expression. BDNF, a critical neuropeptide, is essential for neural signaling, synaptic genesis and plasticity [35, 36]. BDNF, along with its receptors, is implicated in the pathophysiology of neurodegenerative disorders such as Alzheimer's disease, amyotrophic lateral sclerosis, and Rett syndrome, positioning it as a potential therapeutic target [35, 36]. Synthesized in the cytosol of neurons and glial cells, BDNF is distributed throughout the brain, with peak levels in the hippocampus and cortex. It engages numerous signaling pathways that are vital for synaptic function by interacting with TrkB receptors at various neural sites [37]. Correlations have been established between BDNF concentrations and the severity of cognitive impairment [38]. Alterations in BDNF/TrkB signaling have been proposed as a mechanism contributing to cognitive pathological states [39]. Since BDNF is widespread in the nervous system, it can be interfered with by the BDNF-TRKB pathway if EHS patients also have other diseases. For instance, if the patient also suffers from Chronic unpredictable mild stress-induced depression-like behaviors in rats will change the structure of the *Lactobacillus casei* by activating BDNF-TrkB [40]. In addition, the gut microbiota compositions and metabolic fingerprints in mice of the ESH+probiotics group were not determined. Thus, the involvement of gut microbiota composition and bioactive metabolites in these processes could be further explored.

## Conclusion

Our results indicate that cognitive impairment following EHS may be a consequence of thermal injury-induced disruptions in gut microbiota and metabolism, which in turn lead to diminished BDNF expression in the hippocampus via the gut-brain axis. It is suggested that probiotics supplementation potentially upregulates BDNF expression and its related signaling pathways through the gut-brain axis, thereby supporting neural plasticity and mitigating cognitive impairments associated with heat stroke. Although these findings are derived from preliminary animal model research, they necessitate further clinical investigation to determine the efficacy of probiotics in treating cognitive impairments in human cases of EHS.

### Supplementary Information

**Supplementary Material 1.**

## Data Availability

Sequence data that support the findings of this study have been deposited in the European Nucleotide Archive with the primary accession code PRJNA1060907.

## Abbreviations

BDNF: Brain-derived neurotrophic factor
EHS: Exertional Heat Stroke
EPMT: Elevated Plus Maze Test
FMT: Fecal Microbiota Transplantation
FST: Forced Swimming Test
KEGG: Kyoto Encyclopedia of Genes and Genomes
MSEA: Metabolite Sets Enrichment Analysis
NORT: Novel Object Recognition Test
OFT: Open Field Test
O2PLS: Orthogonal projections to latent structures
SCFAs: Short Chain Fatty Acids
TEM: Transmission Electron Microscopy
TrKB: Tropomyosin receptor kinase B
TST: Tail Suspension Test
