# Supplementary material for: Exertional heat stroke-induced changes in gut microbiota cause cognitive impairment in mice
Source: BMC Microbiol. 2024 Apr 23;24:134. doi: 10.1186/s12866-024-03276-7 (PMC11040997; doi:10.1186/s12866-024-03276-7)

TrkB (Expected values: 90/140kD)

(Observed values:97/130kD)


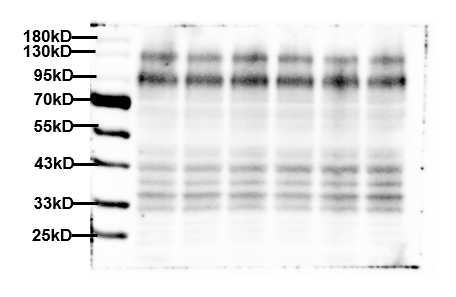


P-TrkB (Expected values: 92kD)

(Observed values:120kD)


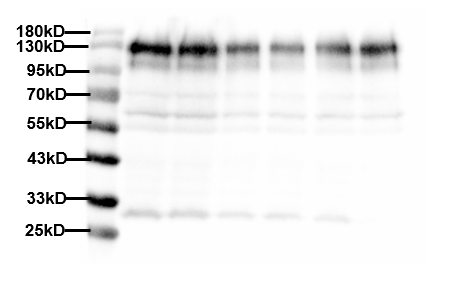


BDNF (Expected values: 37kD)

(Observed values: 43kD)


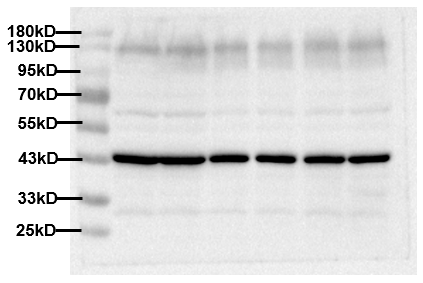


β-actin (Expected values: 43kD)

(Observed values:45kD)


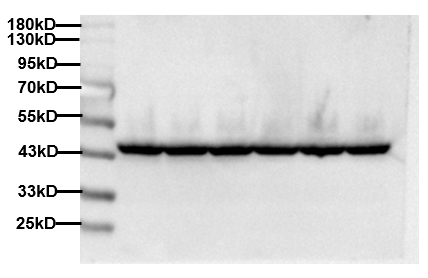

Supplement: Supplementary file 1 — Supplementary Material 1. [file 12866_2024_3276_MOESM1_ESM.docx]
